# Retraction: Prognostic and clinicopathological significance of C-reactive protein/albumin ratio (CAR) in patients with gastric cancer: A meta-analysis

**DOI:** 10.1371/journal.pone.0270727

**Published:** 2022-06-24

**Authors:** The PLOS ONE Editors

After this article was published, similarities were noted between this article and submissions by other research groups which call into question the validity and provenance of the reported results. In light of these issues, the *PLOS ONE* Editors retract this article [1].

JY, HLi, XZ, YZ, DJ, HLu, and JQ did not agree with retraction and stand by the article’s findings.
